# Minor Changes in Erythrocyte Osmotic Fragility in Trace Amine-Associated Receptor 5 (TAAR5) Knockout Mice

**DOI:** 10.3390/ijms22147307

**Published:** 2021-07-07

**Authors:** Ilya S. Zhukov, Larisa G. Kubarskaya, Inessa V. Karpova, Anastasia N. Vaganova, Marina N. Karpenko, Raul R. Gainetdinov

**Affiliations:** 1 Institute of Translational Biomedicine, Saint Petersburg State University, 199034 Saint Petersburg, Russia; ilya.zhukov@skolkovotech.ru (I.S.Z.); anastasia.n.vaganova@gmail.com (A.N.V.); 2 Institute of Experimental Medicine, 197376 Saint Petersburg, Russia; larkub@yandex.ru (L.G.K.); inessa.karpova@gmail.com (I.V.K.); mnkarpenko@mail.ru (M.N.K.); 3 Institute of Toxicology of Federal Medical-Biological Agency, 192019 Saint Petersburg, Russia; 4 Saint Petersburg State University Hospital, Saint Petersburg State University, 199034 Saint Petersburg, Russia

**Keywords:** trace amines, trace amine-associated receptor, TAAR, TAAR5, erythrocyte fragility, hematology, animal knockout model, GPCR

## Abstract

Trace amine-associated receptors (TAARs) are a group of G protein-coupled receptors that are expressed in the olfactory epithelium, central nervous system, and periphery. TAAR family generally consists of nine types of receptors (TAAR1-9), which can detect biogenic amines. During the last 5 years, the TAAR5 receptor became one of the most intriguing receptors in this subfamily. Recent studies revealed that TAAR5 is involved not only in sensing socially relevant odors but also in the regulation of dopamine and serotonin transmission, emotional regulation, and adult neurogenesis by providing significant input from the olfactory system to the limbic brain areas. Such results indicate that future antagonistic TAAR5-based therapies may have high pharmacological potential in the field of neuropsychiatric disorders. TAAR5 is known to be expressed in leucocytes as well. To evaluate potential hematological side effects of such future treatments we analyzed several hematological parameters in mice lacking TAAR5. In these mutants, we observed minor but significant changes in the osmotic fragility test of erythrocytes and hematocrit levels. At the same time, analysis of other parameters including complete blood count and reticulocyte levels showed no significant alterations in TAAR5 knockout mice. Thus, TAAR5 gene knockout leads to minor negative changes in the erythropoiesis or eryptosis processes, and further research in that field is needed. The impact of TAAR5 deficiency on other hematological parameters seems minimal. Such negative, albeit minor, effects of TAAR5 deficiency should be taken into account during future TAAR5-based therapy development.

## 1. Introduction

Trace amines (TA) are heterogeneously distributed endogenous monoamines in the nervous tissue and periphery, which were discovered almost 150 years ago [1]. Their concentration is roughly two orders of magnitude lower in comparison to dopamine, norepinephrine, or serotonin [2]. Despite the low physiological level in tissues (<10 ng/g; 100 nM), TAs have a high rate of metabolism [3]. There are nine subfamilies of trace amine-associated receptors (TAAR1-9) that have been found in mammals, but their roles in physiological processes are little understood [4]. TAAR1 among them is one of the most investigated receptors. Currently, the first TAAR1 agonism-based drugs have entered clinical trials for the treatment of schizophrenia [4,5]. Other TAAR receptors and their ligands may also have the pharmacological potential [5].

Initially, the field of TAAR research has been focused on olfactory and neurotransmission functions. At the same time, there are several reports aimed at understanding the role of TAARs on the periphery. Expression of essentially all TAARs (except TAAR8) was demonstrated with different abundance in five types of isolated human blood leukocytes [6]. Similarly, all TAARs are expressed in the gastrointestinal tract [4] with D-cells expressing high levels of TAAR1 in the gastric epithelium [7]. Moreover, TAAR1 is expressed in the pancreas and can be involved in weight regulation and glucose homeostasis [8]. TAAR1 can be also activated by thyroid hormone metabolites [9] and is presented in a location of thyroid follicles [10] but levels of thyroid hormones were not changed in mice lacking TAAR1 [11,12]. Such data support the hypothesis that TAs and TAARs, in general, can be involved in some physiological processes at the periphery [5,13].

Comparative analysis of hematological and biochemical parameters in mice lacking specific TAARs allows uncovering of new physiological roles of TAARs in the periphery. In previous studies, we observed only minimal alteration in blood parameters of TAAR1 knockout (TAAR1-KO) mice [12]. During a similar investigation in TAAR9-KO rats, we found a significant decrease in total and low-density lipoprotein cholesterol levels under conditions of TAAR9 deficiency [14]. The present study is devoted to the analysis of hematological parameters of TAAR5 knock-out mice. TAAR5 is reported to be expressed in the olfactory system, several brain areas, gastrointestinal tract, and leukocytes [4,5,6]. Recent studies highlighted the significant role of TAAR5 in the central nervous system. Beta-galactosidase mapping of TAAR5 expression showed its localization not only in the glomeruli but also in deeper layers of olfactory bulb projecting to the limbic brain olfactory circuitry [15]. Moreover, TAAR5 knockout mice show increased adult neurogenesis and elevated number of dopamine neurons [16]. Thus, TAAR5 is emerging as a novel target for pharmacology and future TAAR5-based drugs may have the potential for the treatment of neuropsychiatric disorders. Preliminary evaluation of potential safety profile of future TAAR-based therapies by analyzing alterations in knockout animals could provide important cues for directing future preclinical and clinical studies. This is particularly relevant for evaluation of hematological profiles, since expression of TAARs, includingTAAR5, was demonstrated in white blood cells (WBC) both in mice and humans [4,5,6]. Thus, we focused on the evaluation of hematological parameters in wild type (WT), heterozygous TAAR5-KO (HET), and homozygous TAAR5-KO (KO) mice. The following hematological parameters were evaluated: osmotic fragility test (OFT) of erythrocytes, complete blood count (CBC) analysis, and reticulocyte levels.

## 2. Results

### 2.1. Lack of TAAR5 Gene Associated with Increased Osmotic Fragility in Mouse Erythrocytes

The relative amount of hemoglobin released into the supernatant was determined spectrophotometrically at 541, 555, 577 nm wavelengths, with different concentrations of NaCI. Variety in wavelengths allows the detection of differences in the fractional absorption of oxy-hemoglobin and deoxy-hemoglobin [17]. No differences in fractional absorption were observed with three wavelengths demonstrating essentially identical curves. Full results are presented in Table S1 in the Supplemental Information.

**Figure 1:**
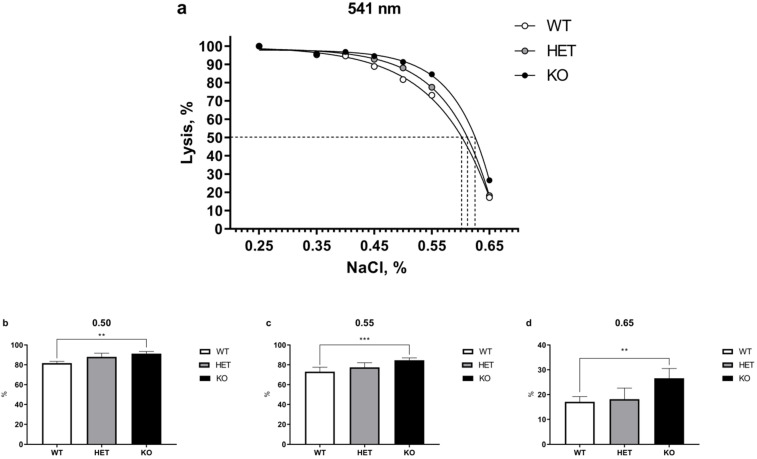
Comparative OFT analysis in the blood of WT, HET, and TAAR5-KO (KO) mice performed at 541 nm wavelength. (**a**) There are significant differences in 0.50, 0.55, and 0.65% NaCl concentration points between KO and WT mice (50% lysis concentration point: WT—0.601, HET—0.612 KO—0.625). (**b**)—Statistical analysis of 0.50% NaCl concentration point revealed significant differences between WT and KO groups. (**c**)—Statistical analysis of 0.55% NaCl concentration point revealed significant differences between WT and KO groups. (**d**)—Statistical analysis of 0.65% NaCl concentration point revealed significant differences between WT and KO groups. Data are mean ± SEM. ** *p* < 0.01, *** *p* < 0.001 vs. WT, two-way ANOVA, post-hoc Tukey test.

The results of OFT test performed at 541 nm wavelength are presented in Figure 1. Two-way ANOVA revealed significant main effect differences between genotypes (*p* < 0.001). Post-hoc Tukey test analysis of WT, HET, and homozygous TAAR5-KO mice demonstrated significant increase in osmotic fragility of erythrocytes between WT and KO groups at certain lysis points. At the same time, there were only minor alterations in 50% lysis points of WT = 0.601, HET = 0.615, and KO = 0.625 mice (Figure 1a).

Figure 1b–d present separate results of significantly changed lysis points at 0.50, 0.55, and 0.65% NaCl between WT and KO groups (0.50: KO = 91.30 ± 2.15% vs. WT = 81.69 ± 1.77%, *p* < 0.01; 0.55: KO = 84.58 ± 2.40% vs. WT = 73.10 ± 4.41%, *p* < 0.001; 0.65: KO = 26.59 ± 3.94% vs. WT = 17.08 ± 2.11%, *p* < 0.01). The parameters of the HET group generally were found between WT and KO groups.

### 2.2. TAAR5 Gene Knockout Does Not Cause Significant Changes in Reticulocyte Levels

**Figure 2:**
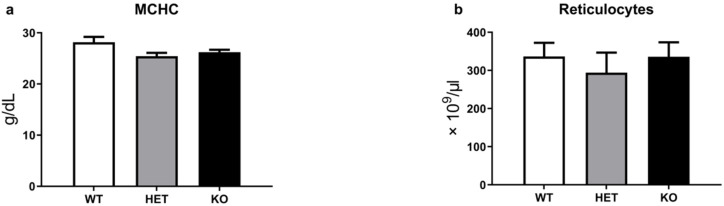
Erythropoiesis parameters of WT, HET, and TAAR5-KO (KO) mice. There are no significant changes in MCHC (**a**) and reticulocytes parameters (**b**). Data are mean ± Standard Error of the Mean (SEM).

In addition to the OFT erythrocyte test, we evaluated mean corpuscular hemoglobin concentration (MCHC) and reticulocyte levels in the blood of WT, HET, and TAAR5-KO mice (Figure 2a,b). In this analysis, we measured reticulocytes via special reagent RETIC solution for Advia2120i (Siemens Healthcare Diagnostics, Eschborn, Germany). Reticulocytes are immature red blood cells and necessary for the evaluation of erythropoiesis processes. There are no significant differences between the three groups in both MCHC and reticulocyte levels.

### 2.3. WBC and Other Routine Hematological Parameters Have Minimal Alterations in Mutant Mice

**Figure 3:**
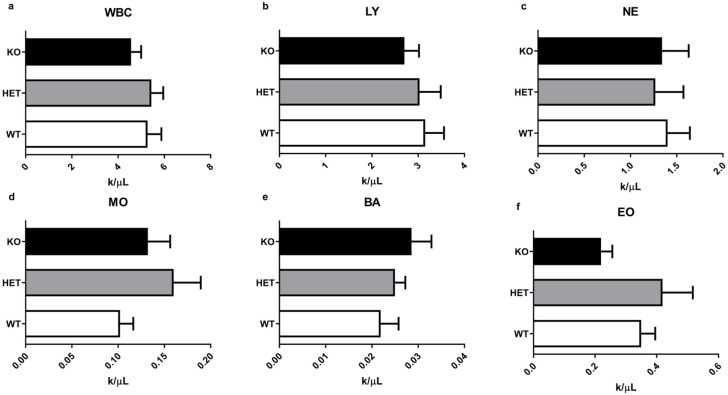

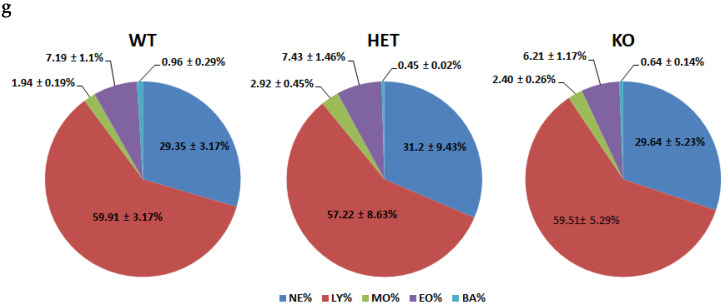
Differentiated WBC parameters in the blood of WT, HET, and TAAR5-KO (KO) mice. Comparative analysis revealed no significant alterations in immune cell parameters: white blood cells (WBC) in total (**a**), lymphocytes (LY) (**b**), neutrophils (NE) (**c**), monocytes (MO) (**d**), basophils (BA) (**e**), eosinophils (EO) (**f**), and leukocyte formula (**g**). Data are mean ± SEM.

**Figure 4:**
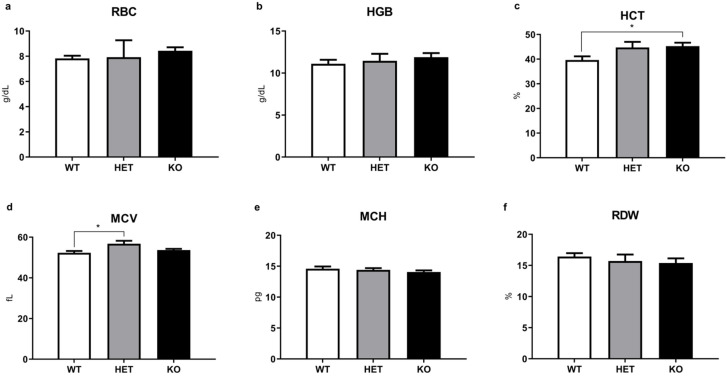
Other routine and additional CBC parameters of WT, HET, and TAAR5-KO (KO) mice. No changes were detected in red blood cells (RBC) (**a**), hemoglobin (HGB) (**b**), mean corpuscular hemoglobin (MCH) (**e**), red blood cell distribution width (RDW) (**f**). Minor but significant alterations were found in hematocrit (HCT) (**c**) and mean corpuscular volume (MCV) (**d**) levels. Data are mean ± SEM. * *p* < 0.05, vs. WT, one-way ANOVA, post-hoc Tukey test.

Further, we investigated if deletion of the TAAR5 gene affects WBC (Figure 3) and other routine CBC parameters (Figure 4). Although there was a minor trend to a higher number of basophils (Figure 3e) there were minimal alterations in WBC parameters. As presented in Figure 3g, there were no significant differences in leukocyte formula between groups.

Analysis of other routine CBC parameters in KO, HET, and WT mice groups (Figure 4) generally revealed minimal differences between groups. However, parameters that are related in part to erythrocyte function [18], hematocrit (HCT), and mean corpuscular volume (MCV), demonstrated a minor increase in mutant mice compared to the WT controls that reached significance in TAAR5-KO group for HCT and TAAR-HET group for MCV (HCT: KO = 44.94 ± 1.45% vs. WT = 39.65 ± 1.48%, *p* < 0.05; MCV: HET = 53.08 ± 0.92 fL vs. WT = 52.21 ± 0.91 fL, *p* < 0.05).

### 2.4. Erythrocyte TAAR5 mRNA Expression in Public Transcriptomic Datasets

Since we observed significant alterations in osmotic fragility in erythrocytes of TAAR5-KO mice we performed analysis of mouse and human TAAR5 mRNA expression in public transcriptomic datasets. Applying the level 0.5 CPM as the threshold, TAAR5 expression was demonstrated in 2 of 4 human erythrocyte specimens in the data set GSE63703. In the GSE128648 the highest TAAR5 expression value was only 0.03 CPM. No TAAR5 expression was identified in human primary adult proerythroblasts. In the murine erythroblasts, TAAR5 expression reaches 1.2–1.4 CPM, but its expression in bone marrow immature erythroid cells was only 0.2–0.3 CPM. Thus the TAAR5 expression seems to vary in erythrocyte maturation which may indicate its involvement in this process.

## 3. Discussion

In this report, we present results of hematological parameters analysis in the blood of TAAR5-KO mice. There are no alterations in most blood parameters, especially in WBC levels, in mutant mice. However, we observed an increased fragility of RBC in the blood of TAAR5-KO mice in OFT test.

Osmotic fragility test (OFT) [19] allows diagnosing erythrocyte abnormalities present in several disorders such as hereditary spherocytosis, autoimmune hemolytic anemia, thalassemia, etc. Such analysis is necessary for revealing hidden pathologies and the preliminary evaluation of the safety profile of medications. The erythrocyte fragility test is a concentration analysis method. It allows measuring the degree and proportion of hemolysis. Low osmotic resistance may lead to intravascular hemolysis, which causes a reduction of the RBC life span [20]. Purified erythrocyte mass is placed in a hypotonic solution of a different medium and subjected to different levels of osmotic stress. Osmotic fragility depends on many factors: the composition of erythrocytes, the integrity of the cell membrane, and the ratio of the surface area to volume. Erythrocyte hemolysis is the process of releasing hemoglobin into plasma due to the rupture of the membrane.

We observed minor but statistically significant changes in osmotic erythrocyte fragility in TAAR5-KO mice. The differences particularly evident at 0.50, 0.55, and 0.65 NaCl (%) concentration points, but 50% lysis points were similar in all genotypes. Furthermore, minor significant alterations were documented in blood parameters that are related in part to erythrocyte function, such as HCT and MCV [18]. It should be noted, however, that both parameters in mutants remain in normal murine ranges [21]. HCT represents the volume percentage of RBCs in total blood and abnormally high levels of HCT are associated with increased risk of cardiovascular disease and high blood viscosity in humans [22]. Erythrocyte abnormalities in hereditary spherocytosis are associated with significantly increased MCHC, RDW, and RBC levels [23]. Increased osmotic erythrocyte fragility in TAAR5-KO mice was not associated with changes in these parameters. Thalassemia-like erythrocyte abnormalities are characterized by nucleated RBC (nRBC) in blood samples [24], but no such pathologies were observed in TAAR5-KO mice (Supplemental Materials, Table S1). The direct clinical indicator in hemolytic anemia is significantly decreased HGB levels [25]. TAAR5-KO animals demonstrated no alterations in HGB levels in comparison to WT mice. Reticulocyte levels also were not affected by the TAAR5 gene deletion. These data indicate that there are likely only minimal alterations in erythropoiesis processes.

Erythrocyte lifespan is 120 days and its starts from the highly regulated process of erythropoiesis [26]. Further, RBC lifespan consists of the following aging stages: spherostomatocyte, stomatocyte, discocyte, echinocyte, spheroechinocyte. Every stage is associated with a specific membrane structure and morphological changes [27]. Finally, echinocytes are degraded via eryptosis process [28]. Intriguingly, a preliminary transcriptomic analysis indicated low expression of TAAR5 in certain datasets from erythrocytes at different stages in both mice and humans. Further detailed studies are needed to evaluate exactly at which stage and how deletion of the TAAR5 gene is causing decreased erythrocyte fragility. Minimal alteration in reticulocyte levels and other hematological parameters suggest that such changes are not connected directly with erythropoiesis processes. Potentially, alterations in eryptosis could contribute to the effects observed. Only 10–20% of erythrocytes are destroyed by intravascular hemolysis, other RBCs are degraded by a wide spectrum of eryptosis triggers in a living organism [29]. One such possibility may be related to the role of TAAR5 receptor as a regulator of monoamine neurotransmitters [15]. Dopamine and epinephrine can inhibit the Ca^2+^-permeable cation channels. As a result, eryptosis can be inhibited by increased levels of catecholamines in the blood [30]. Inhibited eryptosis may lead to increased amounts of old RBC with increased osmotic fragility and HCT levels in the blood. Such a highly regulated system can potentially contribute to altered erythrocyte properties in TAAR5-KO mice. Further detailed studies are necessary to understand the role of TAAR5 in RBC physiology.

In summary, TAAR5 gene knockout causes generally only minimal effect on most hematological parameters despite known expression of TAAR5 in mouse leukocytes [31]. The negative, albeit minor, effect of TAAR5 deficiency on erythrocyte fragility should be taken into account for future TAAR5-based therapy development.

## 4. Materials and Methods

### 4.1. Subject

All animal studies were carried out following the guidelines of the Ministry of Health of the Russian Federation and the principles adopted by the FELASA and RusLASA organizations welfare of laboratory animal use. All experiments were approved by the Saint Petersburg State University Ethical Committee for Animal Research. Littermate WT, HET, and TAAR5-KO mice were derived by crossing heterozygous TAAR5 C57BL6 animals as described [15]. Male mice were used in all experiments. They were housed 3–5/cage, maintained under standard lab conditions (room temperature and humidity were 21 ± 5 °C and 40–70%, respectively), and provided with food and water ad libitum.

Three groups of middle-age (10–12 months old) mice were used in the present study: TAAR5 homozygous knock-out group, (*n* = 14); TAAR5 heterozygous group (*n* = 6) and wild type control group, (*n* = 20). All experiments were conducted during the light phase. All mice were genotyped before and after the experiments.

### 4.2. Sample Collection and Storage Methods

Whole mice trunk blood (postprandial) for CBC and OFT was placed into VACUETTE K3-EDTA tubes (Greiner Bio-One, Kremsmünster, Austria), and stored at +4 °C or room temperature before the experiment (samples were analyzed within 1–3 h after blood collection). After hematological measurement, red blood cells (RBC) of each sample were isolated for the osmotic fragility test.

Serum for biochemical screening (postprandial): rats were shortly anesthetized with isoflurane and killed by decapitation; trunk blood was collected into VACUETTE blood collection tubes for serum (Greiner Bio-One, Kremsmünster, Austria), incubated in a vertical position for 15 min, and then kept at +4 °C until centrifugation. Samples with coagulated blood were centrifuged at 1500 rpm for 15 min at +4 °C. Serum was transferred into dry clean tubes and stored until analysis at −20 °C for no more than 5 days.

### 4.3. Osmotic Fragility Test of Erythrocytes

Mouse sample blood was taken in EDTA tubes and RBC mass was purified. Five microliters of the cell suspension were added later at room temperature to tubes containing 2.5 mL each of graded concentrations of NaCl. (The latter tubes were prepared by diluting the following solution with distilled water to the appropriate salt concentration: 0.25; 0.35; 0.40; 0.45; 0.50; 0.55; 0.65%). The tubes were gently mixed again and after standing 10 min the unlysed red cells were removed by centrifugation.

Normal saline solution for humans is 0.9% (9 g of salt per liter), the minimum limit range of osmotic resistance corresponds to a solution containing 0.42–0.48% NaCl. Complete hemolysis (maximum limit) occurs at a concentration of 0.30–0.34% NaCl. The C57BL mice have decreased RBC fragility compared to a human. As a result, their hemolysis ranges vary between 0.35 and 0.55% NaCl [18]. In the current method, the maximum limits in rats were extended by 0.25–0.65% in case of unpredicted abnormalities.

The relative amount of hemoglobin released into the supernatant was determined spectrophotometrically (Beckmann Coulter DU 800, Carlsbad, CA, USA) at 541, 555, 577 nm, with a 0.85% NaCI sample serving as the blank and a 0.1% sample as the 100% lysis point. Variety in wavelengths allows the detection of differences in the fractional absorption of oxy-hemoglobin and deoxyhemoglobin [19]. All three wavelengths demonstrated almost identical curves. Full results attached in Supplementary Materials, Table S1.

### 4.4. Measurement of Complete Blood Count Parameters

The following hematological tests were performed using an automated hematology analyzer: an ADVIA 2120i (Siemens Healthcare Diagnostics, Eschborn, Germany). This flow cytometry-based system uses light scatter, differential white blood cell (WBC) lysis, and myeloperoxidase, and oxazine 750 stainings to provide a complete blood cell count, a WBC differential, and a reticulocyte count. A cyanide-free method is used to measure hemoglobin colorimetrically. The following hematological CBC parameters were measured: leukocyte subpopulations, erythrocyte count (RBC), hemoglobin, hematocrit (HCT), reticulocytes (Retic), mean corpuscular volume (MCV), mean corpuscular hemoglobin (MCH), mean corpuscular hemoglobin concentration (MCHC), red blood cell distribution width (RDW), platelets (PLT), white blood cells (WBC), neutrophils (NE), lymphocytes (LY), monocytes (MO), eosinophils (EO), and basophils (BA).

### 4.5. Interassay Repeatability

Before analyzing the serum and blood samples, the equipment was decontaminated, calibrated, and checked by internal quality control. Inter-assay repeatability was evaluated by calculating the coefficient of variation (CV) of ten consecutive measurements of internal quality control material in three different controls (low, normal, and high). Coefficients of variation (%) were calculated as standard deviation (SD)/mean × 100.

### 4.6. Statistical Analysis

Values obtained are presented as mean ± SEM and were further subjected to one-way (CBC) and two-way (OFT) analysis of variance (ANOVA) with post-hoc Tukey HSD test, using GraphPad Prism version 6.0 for Windows from GraphPad Software, San Diego, CA, USA. Values of *p* < 0.05 were considered to be significant.

### 4.7. Bioinformatic Analysis of RNA Seq Datasets

Gene Expression Omnibus (GEO) database was searched for datasets corresponding to RNA expression in erythrocytes (search terms “erythrocytes” and “red blood cells”), reticulocytes, or erythroblasts. Datasets for RNA expression in cells were isolated directly from human or mouse blood and were included in the analysis (i.e., GSE108378, GSE63703, GSE128648 for human adult erythrocytes, GSE86910 for human primary adult proerythroblasts, GSE97658 and GSE49843 for murine erythroblasts or bone marrow immature erythroid cells respectively). TAAR5 expression levels in count per million (CPM) values were extracted by the GEO RNA-seq Experiments Interactive Navigator (GREIN, available at: http://www.ilincs.org/apps/grein/?gse=). Following the Expression Atlas thresholds, expression levels 0.5 CPM or higher were interpreted as positive.

## Data Availability

All of the data is presented in the article and supplementary files. No additional data is reported.

## Supplementary Materials

Supplementary materials are available online at https://www.mdpi.com/article/10.3390/ijms22147307/s1.
