# Supplementary material for: Minor Changes in Erythrocyte Osmotic Fragility in Trace Amine-Associated Receptor 5 (TAAR5) Knockout Mice
Source: Int J Mol Sci. 2021 Jul 7;22(14):7307. doi: 10.3390/ijms22147307 (PMC8303310; doi:10.3390/ijms22147307)
Supplement: Supplementary file 1 [file ijms-22-07307-s001.zip › ijms-1263070-SI.pdf]

Supplementary Table S1. Comparative OFT analysis of TAAR5-KO and WT mice.

Data are Mean ± SEM, TAAR5 homozygous knock-out group, (n=13); TAAR5 heterozygous group (n=6) and wild type control group, (n=11).

NaCl	WT										
**0.25**	100.00	100.00	100.00	100.00	100.00	100.00	100.00	100.00	100.00	100.00	100.00
**0.35**	100.00	100.00	92.05	100.00	98.28	84.86	95.23	91.56	89.86	100.00	95.36
**0.4**	93.13	93.64	92.83	99.29	99.68	93.95	94.68	93.43	86.97	100.00	93.61
**0.45**	65.44	92.08	92.00	95.00	85.57	84.38	93.76	90.08	84.20	94.62	100.00
**0.5**	76.52	81.10	86.54	79.55	83.17	75.82	85.44	79.38	76.17	95.72	79.25
**0.55**	71.71	59.47	83.84	41.54	81.85	61.09	88.30	77.36	69.29	91.07	78.66
**0.65**	13.02	18.06	29.24	3.83	13.62	11.28	22.29	14.30	19.26	25.08	17.88

NaCl	HET					
**0.25**	100.00	100.00	100.00	100.00	100.00	100.00
**0.35**	91.99	93.21	91.57	96.97	100.00	100.00
**0.4**	86.91	97.08	96.31	93.68	100.00	100.00
**0.45**	85.43	93.68	93.44	94.17	100.00	89.96
**0.5**	70.83	87.45	91.27	94.41	94.57	89.96
**0.55**	63.37	66.15	79.21	77.11	92.21	86.80
**0.65**	16.14	11.34	16.92	7.87	39.18	17.38

NaCl	KO												
**0.25**	100.00	100.00	100.00	100.00	100.00	100.00	100.00	100.00	100.00	100.00	100.00	100.00	100.00
**0.35**	100.00	100.00	93.12	93.87	86.55	98.79	97.39	94.38	97.85	93.88	96.46	95.53	94.80
**0.4**	100.00	98.30	90.98	99.00	90.44	100.00	100.00	94.93	98.53	97.66	98.89	97.07	92.49
**0.45**	100.00	95.07	87.85	87.32	79.65	98.44	98.62	95.89	94.41	103.03	97.95	94.57	96.61
**0.5**	86.39	90.12	85.79	85.82	71.23	93.76	100.00	93.72	97.06	96.39	93.94	92.95	99.84
**0.55**	85.66	79.44	77.77	72.20	69.50	94.76	89.52	85.09	97.66	89.88	91.39	89.06	77.71
**0.65**	14.80	9.43	11.18	16.54	23.12	31.16	15.38	23.40	40.53	48.48	33.59	54.09	23.95

Supplementary Table S2. Comparative hematological analysis of TAAR5-KO and WT mice. Data are Mean ± SEM, TAAR5 homozygous knock-out group, (n=14); TAAR5 heterozygous group (n=6) and wild type control group, (n=20).

Parameters (units)	WT	HET	KO
RBC(M/ul)	7.8±0.21	8.44±0.27	7.93±0.55
Hgb(g/dL)	11.11±0.91	11.45±1.46	11.89±0.49
HCT(%)	39.65±1.46	44.72±2.32	45.27±1.41
MCV(fL)	52.31±0.91	56.78±1.45	53.7±0.58
MCH(pg)	14.59±0.37	14.4±0.29	14.06±0.26
MCHC(g/dL)	28.16±1.06	25.43±0.65	26.21±0.47
RDW(%)	16.42±0.56	15.7±1.05	15.39±0.76
nRBC(k/ul)	0.00	0.00	0.00
WBC(k/ul)	5.28±0.60	5.44±0.51	4.56±0.44
NE(k/ul)	1.4±0.24	1.27±0.30	1.34±0.29
LY(k/ul)	3.15±0.41	3.03±0.46	2.70±0.32
MO(k/ul)	0.10±0.01	0.16±0.03	0.13±0.03
EO(k/ul)	0.35±0.05	0.42±0.01	0.22±0.04
BA(k/ul)	0.02±0.01	0.03±0.01	0.03±0.01
PLT(k/ul)	1268±60.11	1578±172.4	1285±66.54
MPV(fL)	8.364±0.97	6.917±0.40	7.214±0.51
Retic	336.6±35.98	294.3±52.73	335.8±38.02
LUC	0.52±0.14	0.04±0.01	0.51±0.22
